# ApoE3 vs. ApoE4 Astrocytes: A Detailed Analysis Provides New Insights into Differences in Cholesterol Homeostasis

**DOI:** 10.3390/antiox11112168

**Published:** 2022-11-01

**Authors:** Erica Staurenghi, Valerio Leoni, Marco Lo Iacono, Barbara Sottero, Gabriella Testa, Serena Giannelli, Gabriella Leonarduzzi, Paola Gamba

**Affiliations:** 1 Department of Clinical and Biological Sciences, University of Turin, Orbassano, 10043 Turin, Italy; 2 Laboratory of Clinical Biochemistry, Hospital Pius XI of Desio, ASST-Brianza, University of Milano-Bicocca, 20126 Monza, Italy; 3 Department of Medicine and Surgery, University of Milano-Bicocca, 20126 Monza, Italy

**Keywords:** astrocytes, alzheimer’s disease, apolipoprotein E, ApoE4, cholesterol metabolism, lipids, oxysterols

## Abstract

The strongest genetic risk factor for sporadic Alzheimer’s disease (AD) is the presence of the ε4 allele of the apolipoprotein E (ApoE) gene, the major apolipoprotein involved in brain cholesterol homeostasis. Being astrocytes the main producers of cholesterol and ApoE in the brain, we investigated the impact of the ApoE genotype on astrocyte cholesterol homeostasis. Two mouse astrocytic cell lines expressing the human ApoE3 or ApoE4 isoform were employed. Gas chromatography–mass spectrometry (GC-MS) analysis pointed out that the levels of total cholesterol, cholesterol precursors, and various oxysterols are altered in ApoE4 astrocytes. Moreover, the gene expression analysis of more than 40 lipid-related genes by qRT-PCR showed that certain genes are up-regulated (e.g., CYP27A1) and others down-regulated (e.g., PPARγ, LXRα) in ApoE4, compared to ApoE3 astrocytes. Beyond confirming the significant reduction in the levels of PPARγ, a key transcription factor involved in the maintenance of lipid homeostasis, Western blotting showed that both intracellular and secreted ApoE levels are altered in ApoE4 astrocytes, as well as the levels of receptors and transporters involved in lipid uptake/efflux (ABCA1, LDLR, LRP1, and ApoER2). Data showed that the ApoE genotype clearly affects astrocytic cholesterol homeostasis; however, further investigation is needed to clarify the mechanisms underlying these differences and the consequences on neighboring cells. Indeed, drug development aimed at restoring cholesterol homeostasis could be a potential strategy to counteract AD.

## 1. Introduction

Alzheimer’s disease (AD) is a highly complex neurodegenerative condition that affects millions of people around the world, and the number of AD patients is increasing due to aging of the global population. Two features are the primary hallmarks in the AD brain: intracellular neurofibrillary tangles (NFTs) made of hyperphosphorylated tau protein, and extracellular senile plaques caused by amyloid β (Aβ) accumulation [1].

Most AD cases are sporadic, due to the complex combination of genetic and environmental risk factors. The strongest genetic risk factor for sporadic AD is the presence of the ε4 allele of the apolipoprotein E (ApoE) gene, the major apolipoprotein involved in brain cholesterol transport [2,3]. Indeed, changes in lipid metabolism, in particular that of cholesterol, predispose to AD playing a substantial role in the different phases of the disease [4]. In contrast to the most common isoform ApoE3, present in 78% of the population, ApoE4 increases the risk of developing AD by three to twelve times depending on whether the ε4 allele is present in heterozygosis or homozygosis [3]. Indeed, the ApoE4 isoform has been shown to affect lipid and glucose metabolism, but also many other processes involved in AD pathology including Aβ clearance, neuroinflammation, and synaptic function [5]; however, the molecular mechanisms underlying ApoE4-induced pathogenic processes are not yet clear. In addition to AD, ApoE4 has been associated with the progression of related dementia, such as frontotemporal dementia and Lewy body dementia [6,7].

In humans, the brain is the organ with the highest level of cholesterol, approximately 20% of the whole-body cholesterol pool [8]. Cholesterol homeostasis in the normal brain is tightly regulated in order to keep cholesterol steady-state levels in the brain stable, essential for neuronal functioning and brain development. In the brain most cholesterol is produced de novo by astrocytes, and then transported to neurons and other cells by combining with ApoE, mainly synthesized by astrocytes, to form lipoproteins that are secreted through the ATP-binding cassette (ABC) transporters [9,10]. The association between cholesterol homeostasis alterations and AD onset and progression has been evident for many years [11,12,13]. For instance, cholesterol oxidation products (i.e., oxysterols) that accumulate in the brain are key actors in AD pathogenesis because they lead to neuron dysfunction and degeneration, mainly by enhancing oxidative stress and inflammation [11,14].

Since astrocytes are the main producers of both cholesterol and ApoE in the brain and brain cholesterol homeostasis alterations are closely linked to AD [10], understanding how the ApoE4 genotype affects lipid homeostasis in astrocytes is of primary importance. In this regard, transcriptomic studies of ApoE4 astrocytes deriving from human-induced pluripotent stem cells (hiPSCs) of AD patients demonstrated that lipid metabolism is one of main altered processes [15,16]. Moreover, alterations of lipid metabolism were shown to distinguish ApoE4 astrocytes deriving from human ApoE-targeted replacement (ApoE-TR) mice [17]. These studies clearly highlight the key role of the ApoE4 genotype in affecting lipid homeostasis in astrocytes, but further analyses are needed to clarify its specific impact on cholesterol metabolism and to elucidate the molecular mechanisms involved in the induced metabolic alterations.

To fill this gap, this paper aimed at deepening the impact of ApoE4 genotype on astrocytic cholesterol homeostasis, providing a detailed analysis of the main molecules involved in cholesterol metabolism and transport in ApoE3 and ApoE4 astrocytic cell lines obtained from ApoE-TR mouse primary astrocytes [18]. For the first time, we demonstrated substantial differences between ApoE3 and ApoE4 astrocytes at the level of various molecules involved in cholesterol metabolism and transport. Interestingly, the obtained results pointed out that ApoE4 astrocytes contain altered levels of total cholesterol, cholesterol precursors, and of the main oxysterols. Of note, the trend of the oxysterol concentrations in ApoE4 astrocytes compared to ApoE3 astrocytes was similar to that observed in human AD brain cortices compared to healthy brains [19], suggesting their potential implication in ApoE4 deleterious effects on AD risk.

## 2. Materials and Methods

### 2.1. Cell Cultures

Immortalized mouse astrocytes, obtained from ApoE-TR mouse primary astrocytes expressing human ApoE3 or ApoE4 under the control of endogenous mouse ApoE promoter, were kindly gifted by Prof. David Holtzman [18]. Astrocyte cultures were maintained at 37 °C with 5% CO_2_ in DMEM high glucose medium (Corning, Corning, NY, USA) supplemented with 10% fetal bovine serum (FBS) (Gibco, Thermo Fisher Scientific, Waltham, MA, USA), 4 mM L-Alanyl-L-Glutamine (Glutagro™ Supplement, Corning), 100 U/mL penicillin, and 100 µg/mL streptomycin (Corning). The growing medium was replaced with serum-free medium 24 h before collecting astrocyte-conditioned media (ACM) and cell pellets for protein or RNA extraction.

### 2.2. Sterol Quantification by GC-MS

Cholesterol, cholesterol precursors (lathosterol, lanosterol and desmosterol, markers of cholesterol synthesis), and the oxysterols 25-hydroxycholesterol (25-OHC) and 27-hydroxycholesterol (27-OHC) (markers of reverse cholesterol transport with immunomodulatory and metabolic actions), 24(S)-hydroxycholesterol (24-OHC) (cerebrosterol, brain-specific cholesterol elimination product and marker of brain cholesterol turnover), 7-ketocholesterol (7-KC), 7α-hydroxycholesterol (7α-OHC), and 7β-hydroxycholesterol (7β-OHC) (markers of oxidative stress) were measured by isotope dilution gas chromatography-mass spectrometry (GC-MS), as described elsewhere [20,21,22].

The cell number of each sample (about 20^6^ cells) was evaluated to normalize the corresponding sterol amounts. Cellular homogenates, prepared from pellets suspended in water (250 μL) and sonicated for 15 min, were transferred to a screw-capped vial sealed with a Teflon septum together with 50 μg epicoprostanol (Sigma-Aldrich, Merck, Darmstadt, DE, Germany), 500 ng of lathosterol-25, 26, 26, 26, 27, 27, 27-d7, 50 ng of lanosterol-26, 26, 26, 27, 27, 27-d6, 50 ng of 7β-OHC-25, 26, 26, 26, 27, 27, 27-d7, 50 ng of 7-KC-25, 26, 26, 26, 27, 27, 27-d7, 50 ng of 24(R/S)-OHC-25, 26, 26, 26, 27, 27, 27-d7, 50 ng of 25-OHC-26, 26, 26, 27, 27, 27-d6, 50 ng of 27-OHC-25, 26, 26, 26, 27, 27-d6 (Avanti Polar Lipids Inc., Birmingham, AL, USA) as internal standards, as well as 50 μL of butylated hydroxytoluene (BHT) (5 g/L, Sigma-Aldrich) and 50 μL of K3-ethylenediamine tetra acetic acid (EDTA) (10 g/L, Sigma-Aldrich) to prevent autooxidation. Each vial was flushed with argon for 10 min to remove air.

Alkaline hydrolysis was carried out at room temperature for 1 h in the presence of ethanolic KOH 1M. Sterols and oxysterols were extracted twice with 5 mL of cyclohexane followed by centrifugation (3500× *g* for 10 min at 4 °C). The organic solvents were evaporated under a gentle stream of argon and converted into trimethylsilyl ethers with 100 μL N,O-bis(trimethylsilyl)trifluoroacetamide (BSTFA) (Sigma-Aldrich) (70 °C for 60 min).

Isotope dilution GC-MS analysis was performed by a 6890N Network GC system (Agilent Technologies, Santa Clara, CA, USA) equipped with an HP 7687 series autosampler and a HP 7683 series injector (Agilent Technologies) and coupled to a quadruple mass selective detector HP5975B Inert MSD (Agilent Technologies). For GC separation a B-XLB column (30 m × 0.25 mm i.d. × 0.25 μm film thickness; J&W Scientific Alltech, Folsom, CA, USA) was used and the following oven temperature program: an initial temperature of 180 °C held for 1 min, followed by a linear ramp of 20 °C/min to 270 °C, and then a linear ramp of 5 °C/min until the final temperature of 290 °C held for 11 min. Helium was used as a carrier gas at a flow rate of 1 mL/min. Injection of the sample (1 μL) was carried out in splitless mode, at 250 °C with a flow rate of 20 mL/min. The MS temperature parameters were 290 °C for the transfer line, 150 °C for the filament, and 220 °C for the quadrupole, according with the manufacturer indication.

Mass spectrometric data were acquired in selected ion monitoring mode (OTMSi-ethers) at *m/z* = 465 for lathosterol-d7, *m/z* = 458 for lathosterol, *m/z* = 343 for desmosterol, *m/z* = 399 for lanosterol-d6, *m/z* = 393 for lanosterol, *m/z* = 463 for 7α-OHC-d7, *m/z* = 456 for 7α-OHC, *m/z* = 463 for 7β-OHC-d7, *m/z* = 456 for 7β-OHC, *m/z* = 479 for 7-KC-d7, *m/z* = 472 for 7-KC, *m/z* = 137 for 25-OHC-d6, *m/z* = 131 for 25-OHC, *m/z* = 420 for 24-OHC-d7, *m/z* = 413 for 24(S)-OHC, *m/z* = 462 for 27-OHC-d6, and *m/z* = 456 for 27-OHC (Avanti Polar Lipids Inc.). Peak integration was performed manually, and the analytes were quantified from SIM analysis against internal standards using standard curves for the listed compounds.

### 2.3. RNA Extraction and Real-Time RT-PCR

Total RNA was extracted by using TRIzol™ Reagent (Invitrogen, Thermo Fisher Scientific) following the manufacturers’ instructions. Possible traces of DNA were removed by using the TURBO DNA-free™ Kit (Invitrogen) and RNA was dissolved in RNase-free water with RNase inhibitors (SUPERase-In RNase Inhibitor, Invitrogen). The amount and purity of the extracted RNA were assessed spectrophotometrically. The cDNA was synthesized by reverse transcription of 4 μg of RNA by using a commercial kit and random primers (High-Capacity cDNA Reverse Transcription Kit, Applied Biosystems, Thermo Fisher Scientific).

Real-time RT-PCR was performed on 50 ng of cDNA by using a 7500 Fast Real-Time PCR System and the TaqMan Array Mouse Lipid Regulated Genes 96-well Plate or TaqMan Gene Expression Assays for mouse CYP7A1 (Mm00484150_m1), CYP27A1 (Mm00470430_m1), CYP46A1 (Mm00487306_m1), CH25H (Mm00515486_s1), DHCR24 (Mm00519071_m1), HMGCR (Mm01282499_m1), and GAPDH (Mm99999915_g1) (Applied Biosystems). The PCR cycling parameters were set up as previously described [19]. The fractional cycle number (Ct) was determined for each considered gene. Gene expression analysed by using the TaqMan Array Mouse Lipid Regulated Genes 96-well Plate was normalized to the average expression levels of three endogenous control genes included in the plate: glyceraldehyde-3-phosphate dehydrogenase (GAPDH), hypoxanthine phosphoribosyltransferase 1 (HPRT1), and β-glucuronidase (GUSB). The expression levels of two of the 43 genes included in the plate were not calculated because they were undetectable in both ApoE3 and ApoE4 astrocytic cell lines. Gene expression results obtained by using single TaqMan Gene Expression Assays were normalized to the GAPDH expression levels. Relative quantification of target gene expression was achieved by a mathematical method [23].

### 2.4. Gel Electrophoresis and Western Blotting

After incubating astrocytes with serum-free medium for 24 h, ACM were collected, centrifuged at 3000 rpm for 5 min, and a protease inhibitor cocktail (P8340, Sigma-Aldrich) was added before freezing them. To detect secreted ApoE, 3 mL of ACM were concentrated by using centrifugal filters (Amicon Ultra, Millipore, Merck). Astrocytes were washed with phosphate-buffered saline (PBS), pelleted, and lysed with ice-cold PBS containing 10 μL/mL Triton-X100, 10 μL/mL SDS 10%, protease inhibitor (Complete Mini EDTA-Free Protease Inhibitor Cocktail, Roche, Basel, CH) and phosphatase inhibitor (P0044, Sigma-Aldrich) cocktails. Total protein concentration of culture media and cell lysates was determined by Bradford assay (Bio-Rad Protein Assay Dye Reagent Concentrate, Bio-Rad Laboratories, Hercules, CA, USA).

Equal amounts of protein samples (40–100 μg) were mixed with sample buffer (NuPAGE LDS Sample Buffer 4X, Invitrogen) and reducing agent (NuPAGE Sample Reducing Agent 10X, Invitrogen) before boiling them. Then, protein samples were separated by electrophoresis by using 10% acrylamide gels (TGX Stain-Free FastCast Acrylamide kit, Bio-Rad Laboratories) or 4–12% precast gels (Bolt Bis-Tris Plus gels, Invitrogen) and transferred to nitrocellulose membranes (Amersham Protran, GE Healthcare, Chicago, IL, USA). After blocking with 5% non-fat dry milk in Tris-buffered saline (TBS) for 1 h at room temperature, membranes were incubated with primary antibodies overnight at 4 °C. The following primary antibodies (1:1000 dilution) were used: anti-ApoE (D7I9N, Cell Signaling Technology, Danvers, MA, USA), anti-PPARγ (C26H12, Cell Signaling Technology), anti-ABCA1 (MA5-16026, Invitrogen), anti-ApoER2 (LRP8, PA5-109269, Invitrogen), anti-LDLR (MA5-32075, Invitrogen), anti-LRP1 (37-7600, Invitrogen), and anti-β-actin (sc-47778, Santa Cruz Biotechnology, Dallas, TX, USA). After washing with TBS1X-Tween20 0.05% to remove the unbound antibody, the appropriate HRP-linked secondary antibody (1:3000, Cell Signaling Technology) was added for 1 h at room temperature. Membranes were washed with TBS1X-Tween20 0.05%, incubated with Clarity Western ECL Substrate (Bio-Rad Laboratories) and scanned by using a ChemiDoc Imaging System (Bio-Rad Laboratories). Band intensities were quantified by using the Image Lab Software (Bio-Rad Laboratories) and normalized to the corresponding β-actin or, in the case of concentrated media samples, to the total protein content of the sample through the Stain-Free imaging technology (Bio-Rad Laboratories).

### 2.5. Immunocytochemistry

Astrocytes were plated on coverslips (16 mm diameter, thickness No. 1) in 12-well plates. After 24 h in serum-free medium, cells were washed with PBS, fixed in 4% paraformaldehyde in PBS for 20 min at room temperature, and then washed again twice with PBS. Cells were permeabilized and blocked (2% goat serum, 0.1% Triton in PBS) for 1 h at room temperature before being incubated with a mix of anti-ApoE (1:100, D7I9N, Cell Signaling Technology) and anti-β-actin (1:50, sc-47778, Santa Cruz Biotechnology) primary antibodies for 2 h at room temperature. After two washes in PBS, astrocytes were incubated with a mix of Alexa Fluor 594 (1:250, ab150080, Abcam, Cambridge, UK) and Alexa Fluor 488 (1:250, A11001, Invitrogen) secondary antibodies for 1 h at room temperature; then, nuclei were stained with 4’,6-diamidino-2-phenylindole (DAPI) (2 μg/mL in PBS, Sigma-Aldrich) for 30 min at room temperature. Cells were imaged by using an LSM800 confocal microscope (Carl Zeiss, Oberkochen, DE, Germany).

### 2.6. Statistical Analysis

Data were analysed by using Student’s *t*-test with Welch correction (GraphPad Prism 7 Software, Graphpad Software, La Jolla, CA, USA). Results were considered statistically significant when *p* < 0.05. Data are represented as means ± standard deviation (SD).

## 3. Results

### 3.1. ApoE Intra- and Extracellular Levels Are Altered in ApoE4 Astrocytes

**Figure 1:**
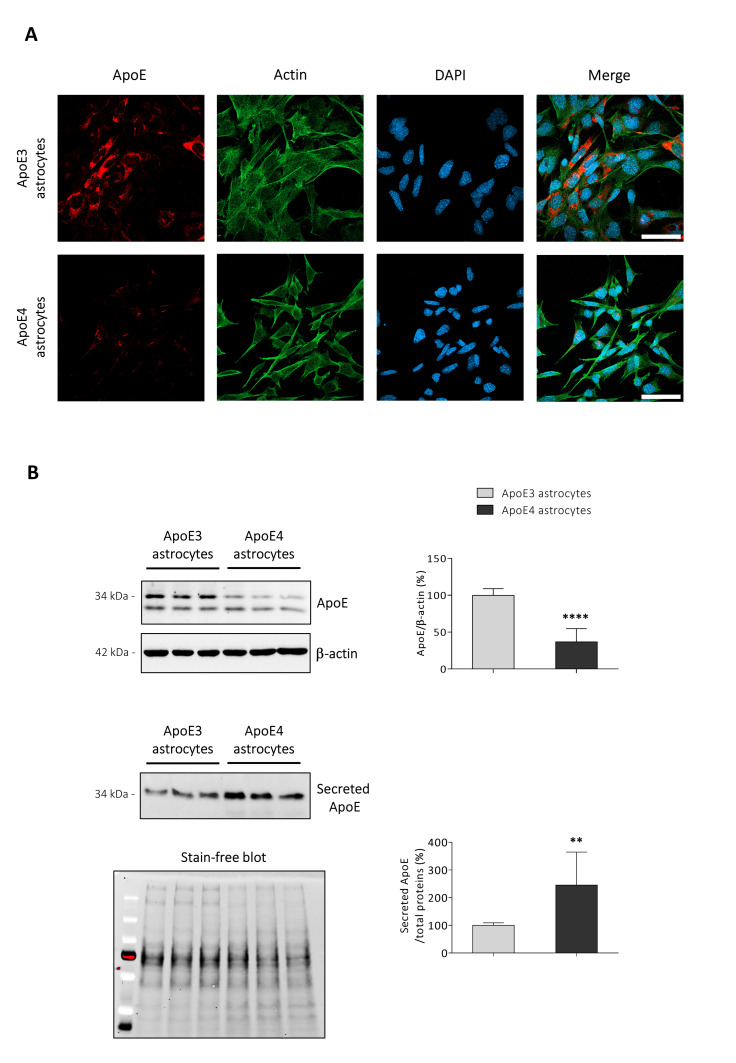
ApoE synthesis and release is altered in ApoE4 astrocytes. (**A**) ApoE protein levels were examined by immunocytochemistry. Antibodies detecting ApoE (red), and β-actin (green), were used and nuclei were stained with DAPI (blue). Representative images are shown. Cells were imaged by using an LSM800 confocal microscope (Zeiss, 63X oil objective; scale bar: 50 μm). (**B**) ApoE protein levels were analysed by Western blotting in both cell lysates and concentrated media from ApoE3 and ApoE4 immortalized mouse astrocytes. Concerning cell lysates, ApoE levels were normalized to the corresponding β-actin levels and data are represented as percentages of average ApoE3 values. Data are expressed as mean values ± SD of four different experiments (*n* = 14, Student’s *t*-test). Concerning concentrated media, ApoE levels were normalized to total proteins loaded into the corresponding well (Stain-free method) and data are represented as percentage of average ApoE3 values. Data are expressed as mean values ± SD of four different experiments (*n* = 11, Student’s *t*-test). **** *p* < 0.0001, ** *p* < 0.01 vs. ApoE3 astrocytes.

Alterations of ApoE synthesis and release have been shown to characterize hiPSCs-derived ApoE4 astrocytes [15,16,24]. Results confirmed that the ApoE intracellular content in immortalized ApoE4 astrocytes is less than halved compared to the ApoE amount present in ApoE3 astrocytes, as shown by both immunocytochemistry (Figure 1A) and Western blotting (*p* < 0.0001; Figure 1B). On the other hand, the levels of ApoE released in ACM by the ApoE4 cell line resulted to be more than doubled compared to that secreted by the ApoE3 cell line (*p* < 0.01; Figure 1B).

### 3.2. ApoE4 Astrocytes Show Different Expression Levels of Several Lipid-Related Genes

**Figure 2:**
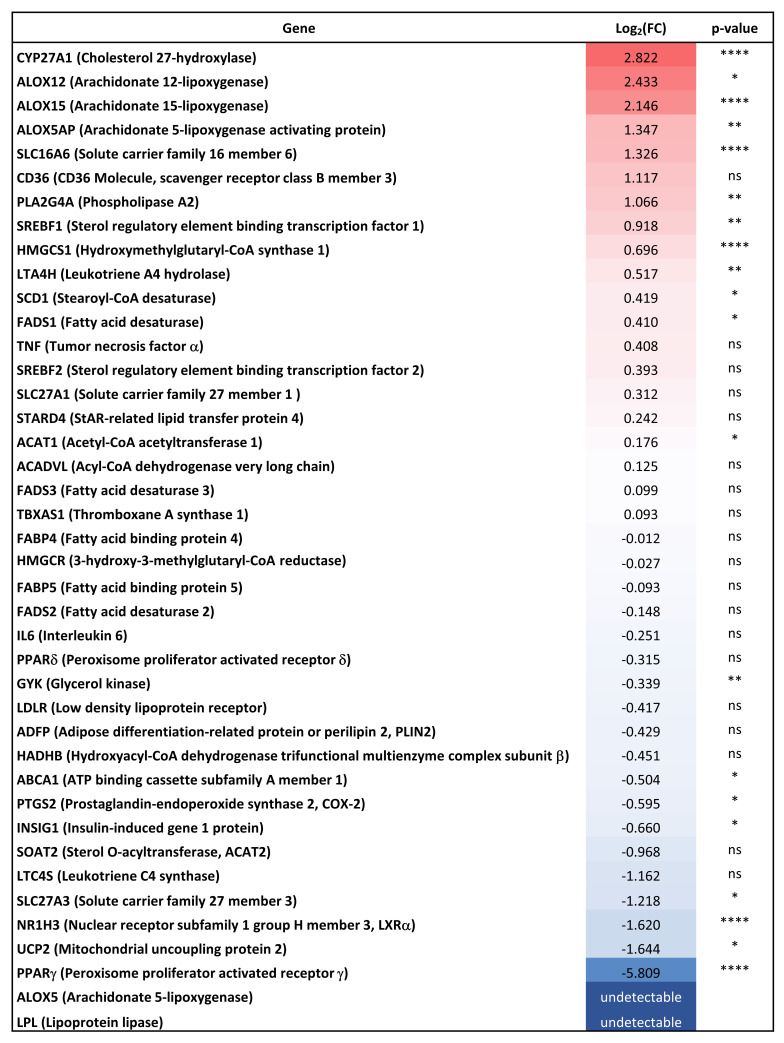
Gene expression analysis of lipid-related genes. Gene expression levels of 43 lipid-related genes were analysed in ApoE3 and ApoE4 immortalized mouse astrocytes by using the TaqMan Array Mouse Lipid Regulated Genes 96-well Plate. The Figure shows the expression levels of each gene in ApoE4 astrocytes (up-regulated genes: red; down-regulated genes: blue) compared to ApoE3 astrocytes. Gene expression results were normalized to the average expression levels of three endogenous control genes included in the plate: glyceraldehyde-3-phosphate dehydrogenase (GAPDH), hypoxanthine phosphoribosyltransferase 1 (HPRT1), and β-glucuronidase (GUSB). Arachinodate 5-lipoxygenase (ALOX5) and lipoprotein lipase (LPL) expression levels were undetectable in ApoE4 astrocytes, so it was not possible to calculate the Log_2_(FC) of these genes. Data are expressed as mean values of three different experiments (*n* = 6, Student’s *t*-test). **** *p* < 0.0001, ** *p* < 0.01, and * *p* < 0.05 vs. ApoE3 astrocytes; ns: not significant.

Transcriptomic studies showed that lipid metabolism is one of the main altered processes in ApoE4 astrocytes [15,16]. In order to investigate this aspect, the expression levels of 43 lipid-related genes were analysed in both ApoE3 and ApoE4 astrocytes and several genes resulted to be up- or down-regulated in ApoE4 compared to ApoE3 astrocytes (Figure 2).

Of the genes that were found significantly more expressed in ApoE4 astrocytes, starting from the most up-regulated one, are those coding for cholesterol 27-hydroxylase (CYP27A1; *p* < 0.0001), arachidonate 12-lipoxygenase (ALOX12; *p* < 0.05), arachidonate 15-lipoxygenase (ALOX15; *p* < 0.0001), arachidonate 5-lipoxygenase activating protein (ALOX5AP; *p* < 0.01), solute carrier family 16 member 6 (SLC16A6; *p* < 0.0001), phospholipase A2 (PLA2G4A; *p* < 0.01), sterol regulatory element binding transcription factor 1 (SREBF1; *p* < 0.01), hydroxy-methylglutaryl-CoA synthase 1 (HMGCS1; *p* < 0.0001), leukotriene A4 hydrolase (LTA4H; *p* < 0.01), stearoyl-CoA desaturase (SCD1; *p* < 0.05), fatty acid desaturase (FADS1; *p* < 0.05), and acetyl-CoA acetyltransferase 1 (ACAT1; *p* < 0.05).

On the other hand, the most down-regulated genes in ApoE4 astrocytes are peroxisome proliferator activated receptor γ (PPARγ; *p* < 0.0001), mitochondrial uncoupling protein 2 (UCP2; *p* < 0.05), nuclear receptor subfamily 1 group H member 3 (NR1H3, also known as LXRα; *p* < 0.0001), solute carrier family 27 member 3 (SLC27A3; *p* < 0.05), insulin-induced gene 1 protein (INSIG1; *p* < 0.05), prostaglandin-endoperoxide synthase 2 (PTGS2, also known as COX-2; *p* < 0.05), ABC subfamily A member 1 (ABCA1; *p* < 0.05), and glycerol kinase (GYK; *p* < 0.01). Notably, the expression levels of lipoprotein lipase (LPL) and arachidonate 5-lipoxygenase (ALOX5) were found to be down-regulated but undetectable in ApoE4 astrocytes, indicating the almost total absence of expression of these genes. Thus, it was not possible to calculate the exact extent and significance of these down-regulations. The expression levels of ABCG1 and interleukin 1β (IL1β) are not reported because they were undetectable in both cell lines.

### 3.3. ApoE4 Astrocytes Are Characterized by a Lower Cholesterol Synthesis

**Figure 3:**
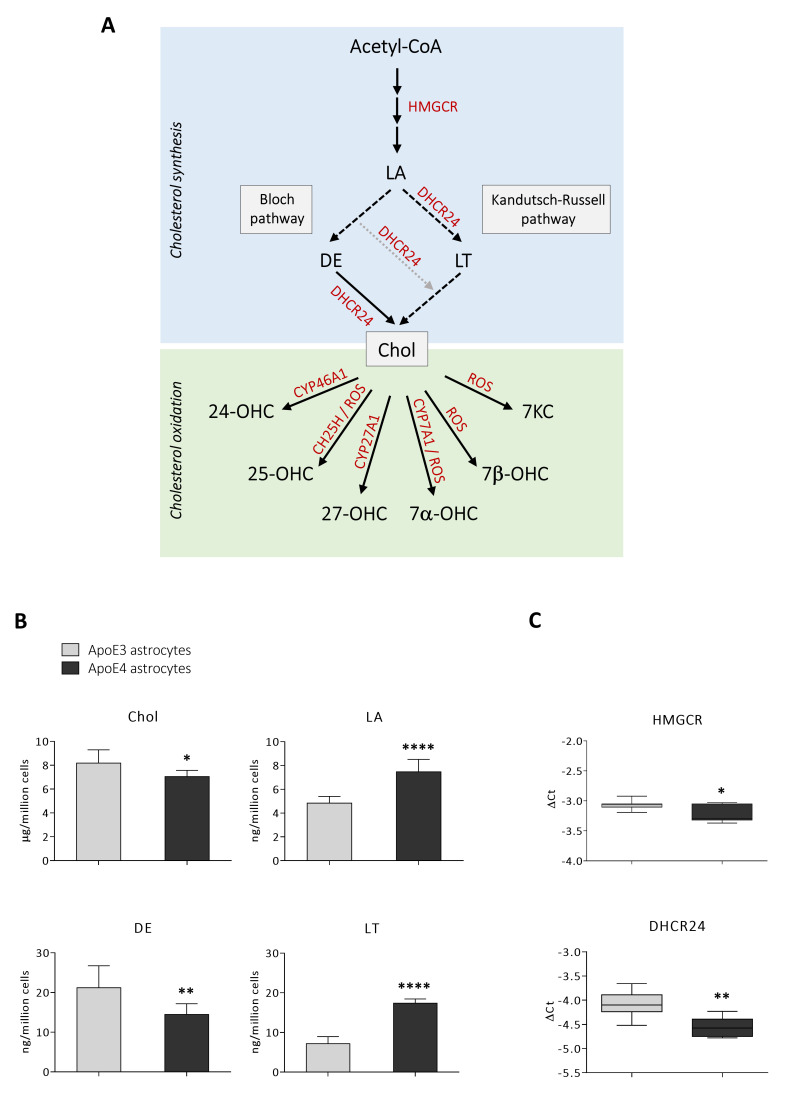
Quantification of cholesterol and its precursors, and analysis of key enzymes expression. (**A**) Schematic representation of cholesterol synthesis and oxidation. Acetyl-CoA: Acetyl coenzyme A; Chol: cholesterol; CH25H: cholesterol 25-hydroxylase; CYP46A1: cholesterol 24-hydroxylase; CYP27A1: cholesterol 27-hydroxylase; CYP7A1: cholesterol 7α-hydroxylase; DE: desmosterol; DHCR24: 24-dehydrocholesterol reductase; HMGCR: 3-hydroxy-3-methylglutaryl-CoA reductase; LA: lanosterol; LT: lathosterol; ROS: reactive oxygen species; 7-KC: 7-ketocholesterol; 7α-OHC: 7α-hydroxycholesterol; 7β-OHC; 7β-hydroxycholesterol; 24-OHC: 24-hydroxycholesterol; 25-OHC: 25-hydroxycholesterol; 27-OHC: 27-hydroxycholesterol. (**B**) Total cholesterol and cholesterol precursors (LA, LT, and DE) were quantified by gas chromatography-mass spectrometry (GC-MS) in ApoE3 and ApoE4 immortalized mouse astrocytes. Data are expressed as mean values ± SD of three different experiments (*n* = 9, Student’s *t*-test). **** *p* < 0.0001, ** *p* < 0.01, and * *p* < 0.05 vs. ApoE3 astrocytes. (**C**) Gene expression levels of the enzymes HMGCR and DHCR24 were analysed in ApoE3 and ApoE4 immortalized mouse astrocytes. Gene expression results were normalized to GAPDH expression levels. Data are expressed as mean values of three different experiments (*n* = 8, Student’s *t*-test). ** *p* < 0.01, and * *p* < 0.05 vs. ApoE3 astrocytes.

It has previously been shown that cholesterol homeostasis is impaired in ApoE4 astrocytes; however, results are still controversial, and it seems that they depend on the specific experimental model [15,16,24]. Thus, we quantified total cholesterol and some biosynthetic cholesterol precursors by GC-MS to have a broader view of the lipid profile of the ApoE3 and ApoE4 cell lines. A simplified representation of cholesterol synthesis and oxidation is depicted in Figure 3A. The analysis pointed out that the levels of the cholesterol precursors lanosterol (+54%; *p* < 0.0001) and lathosterol (+142%; *p* < 0.0001) are higher in ApoE4 astrocytes, whereas desmosterol levels are lower (−32%; *p* < 0.01) compared to ApoE3 astrocytes. Overall, total cholesterol levels are slightly but significantly reduced (−14%; *p* < 0.05) (Figure 3B). Moreover, we analysed the gene expression levels of two key enzymes involved in cholesterol synthesis, 3-hydroxy-3-methylglutaryl-CoA reductase (HMGCR) and 24-dehydrocholesterol reductase (DHCR24). As shown in Figure 3C, HMGCR (*p* < 0.05) and DHCR24 (*p* < 0.01) expression levels are significantly reduced in ApoE4 astrocytes.

### 3.4. Oxysterol Profile Is Different in ApoE4 Astrocytes

**Figure 4:**
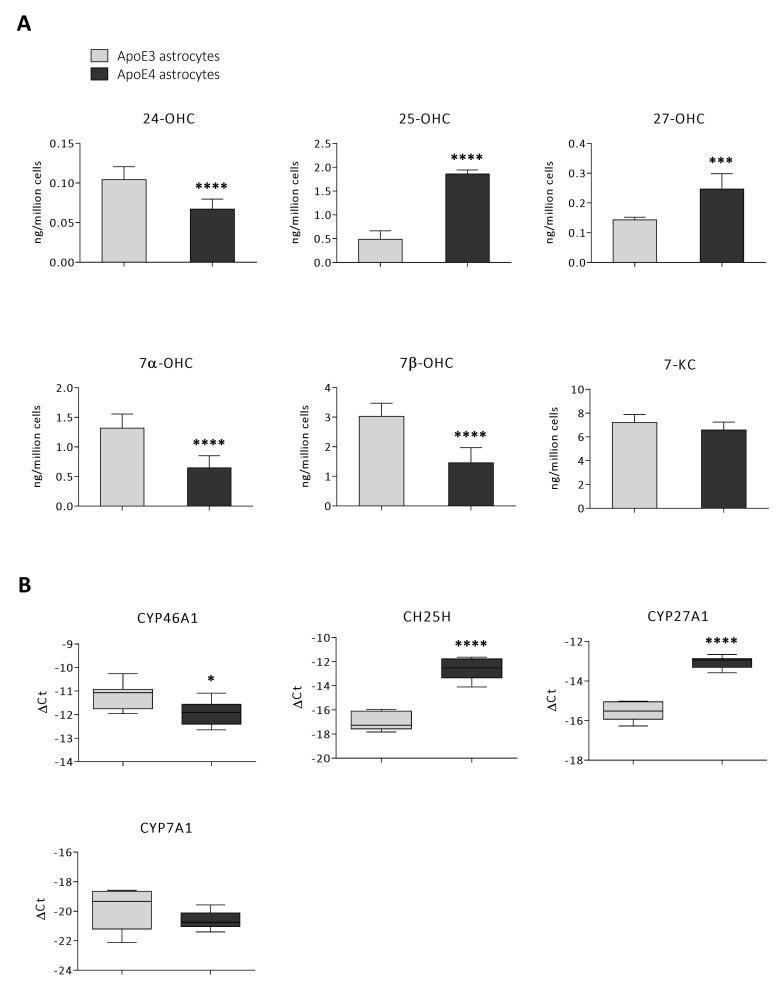
Quantification of oxysterols and gene expression analysis of the enzymes involved in cholesterol oxidation. (**A**) The amount of the oxysterols 24-hydroxycholesterol (24-OHC), 25-hydroxycholesterol (25-OHC), 27-hydroxycholesterol (27-OHC), 7α-hydroxycholesterol (7α-OHC), 7β-hydroxycholesterol (7β-OHC), and 7-ketocholesterol (7-KC) were quantified by gas chromatography-mass spectrometry (GC-MS) in ApoE3 and ApoE4 immortalized mouse astrocytes. Data are expressed as mean values ± SD of three different experiments (*n* = 9, Student’s *t*-test). **** *p* < 0.0001, *** *p* < 0.001 vs. ApoE3 astrocytes. (**B**) Gene expression levels of the enzymes cholesterol 24-hydroxylase (CYP46A1), cholesterol 25-hydroxylase (CH25H), cholesterol 27-hydroxylase (CYP27A1), and cholesterol 7α-hydroxylase (CYP7A1) were analysed in ApoE3 and ApoE4 immortalized mouse astrocytes. Gene expression results were normalized to GAPDH expression levels. Data are shown either as Log_2_(gene/GAPDH) or as Log_2_(FC). Data are expressed as mean values ± SD of three different experiments (*n* = 8, Student’s *t*-test). **** *p* < 0.0001, * *p* < 0.05 vs. ApoE3 astrocytes.

Since AD is associated with abnormalities not only in cholesterol biosynthesis but also in its catabolism by oxidation [11,19,25], we also analysed oxysterol levels in ApoE3 and ApoE4 astrocytes by GC-MS. Concerning the oxysterols of enzymatic origin, ApoE4 astrocytes have been shown to produce higher levels of 25-OHC (+282%; *p* < 0.0001) and 27-OHC (+72%; *p* < 0.001), whereas cholesterol oxidation to 24-OHC (−36%; *p* < 0.0001) and 7α-OHC (−51%; *p* < 0.0001) resulted in being lower. Among the oxysterols of non-enzymatic origin, 7β-OHC levels were found to be lower (−52%; *p* < 0.0001) and 7-KC levels did not change significantly in ApoE4 astrocytes (Figure 4A). In order to better understand the obtained results concerning oxysterol levels, we analysed the gene expression levels of the enzymes responsible for the synthesis of the quantified enzymatically produced oxysterols. In particular, the latter are the enzymes cholesterol 24-hydroxylase (CYP46A1), cholesterol 25-hydroxylase (CH25H), CYP27A1, and cholesterol 7α-hydroxylase (CYP7A1), respectively, responsible for the synthesis of 24-OHC, 25-OHC, 27-OHC, and 7α-OHC, as outlined in Figure 3A. Specifically, their expression levels resulted in reflecting the corresponding oxysterol trend; indeed, CH25H (*p* < 0.0001) and CYP27A1 (*p* < 0.0001) gene expression levels markedly increased in ApoE4 astrocytes, whereas those of CYP46A1 (*p* < 0.05) resulted to be reduced compared to ApoE3 astrocytes. Instead, CYP7A1 expression did not significantly change (Figure 4B).

### 3.5. ApoE4 Astrocytes Synthetize Lower Amounts of KEY Proteins Involved in Cholesterol Homeostasis

**Figure 5:**
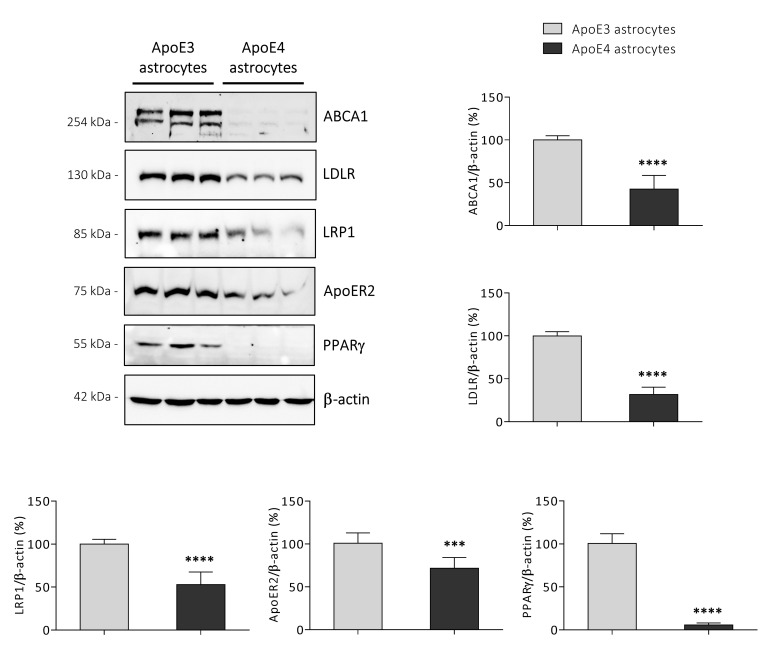
Analysis of the levels of key proteins involved in lipid homeostasis. The protein levels of the ATP binding cassette subfamily A member 1 (ABCA1), ApoE receptor 2 (ApoER2), low-density lipoprotein receptor (LDLR), LDLR-related protein 1 (LRP1), and peroxisome proliferator activated receptor γ (PPARγ) were determined by Western blotting in ApoE3 and ApoE4 immortalized mouse astrocytes. Data were normalized to the corresponding β-actin levels and are shown as percentage of average ApoE3 values. Data are expressed as mean values ± SD of three different experiments (*n* = 9, Student’s *t*-test). **** *p* < 0.0001 and *** *p* < 0.001 vs. ApoE3 astrocytes.

In order to better characterize the ApoE4 astrocytic cell line, we analysed the levels of key proteins involved in the regulation of cholesterol homeostasis in both ApoE3 and ApoE4 cell lines. We observed that ApoE4 astrocytes are characterized by substantial lower levels of proteins involved in cholesterol efflux and uptake, i.e., the transporter ABCA1 (*p* < 0.0001) and the receptors low-density lipoprotein receptor (LDLR; *p* < 0.0001), LDLR-related protein 1 (LRP1; *p* < 0.0001), and ApoE receptor 2 (ApoER2; *p* < 0.001) (Figure 5). In addition, data confirmed that PPARγ protein levels are markedly decreased in ApoE4 compared to ApoE3 astrocytes (*p* < 0.0001) (Figure 5), as suggested by its very low gene expression levels (Figure 2).

## 4. Discussion

ApoE is the primary apolipoprotein responsible for the transport of cholesterol in the brain, where astrocytes are its main producers [26]. After ApoE secretion and lipidation, ApoE-containing lipoproteins are taken up by neurons by binding to different types of receptors, playing a key function in neuronal maintenance and repair (e.g., synaptic homeostasis and axonal regeneration) [27,28]. Since ApoE4 has been identified as the strongest genetic risk factor for sporadic AD, more and more studies have been carried out in order to understand the structural and functional consequences due to the single amino acid change that characterizes ApoE4 (Arg112) compared to the most common ApoE3 isoform (Cys112) [29]. Even if Arg112 is not part of the LDLR binding site, studies showed that ApoE4 has different specificities in binding lipids, and the lipidation status is assumed to affect its ability to bind receptors [30,31]. However, the current knowledge about ApoE role in AD pathology is still limited because native and lipidated ApoE forms present in vivo have not yet been analysed, due to the complexity of the structures and to the insufficient methods currently available [29]. Concerning ApoE4 brain implications, ApoE4 carriers showed an increased Aβ deposition [32,33], a greater hippocampal and cortical atrophy [34], and a higher risk of progression from mild cognitive impairment to AD [35]. Moreover, many of these features also characterize ApoE4-TR mice, which showed cognitive impairment [36,37], Aβ and hyperphosphorylated tau accumulation [38], and deficits in synaptic transmission [39].

Although the role of ApoE4 in AD onset and progression remains unclear, many studies pointed out that ApoE4 may contribute to AD pathogenesis affecting neuronal structure and function, Aβ production and clearance, tau pathology, and neuroinflammation [40]. In particular, new mouse models, in vitro models (e.g., derived from hiPSC), and genome editing technics allowed to a deeper understanding of the impact of ApoE4 in different brain cell types, including astrocytes [41]. ApoE4 has been shown to impact on many physiological functions played by astrocytes, affecting their ability to clear Aβ, to uptake glutamate, to promote neuronal synaptogenesis, and to maintain lipid homeostasis [15,16,42]. Among all these aspects, being astrocytes the main producers of cholesterol and ApoE in the brain, we decided to deepen the impact of ApoE4 genotype on astrocytic cholesterol homeostasis in order to better characterize the induced metabolic and molecular alterations. For this purpose, ApoE3 and ApoE4 astrocytic cell lines obtained from ApoE-TR mouse primary astrocytes were used as experimental models [18]. As shown in Figure 1A, the two cell lines have a different morphology, with ApoE4 astrocytes resulting as smaller compared to ApoE3 astrocytes. Morphological differences have been also observed by other researchers in hiPSC-derived ApoE4 astrocytes, which showed a reduced expression of genes involved in the regulation of actin cytoskeleton and focal adhesions, as well as a reduced attached cell surface area [15]. Concerning ApoE levels, we observed that ApoE intracellular levels in ApoE4 astrocytes are less than half compared to the levels detected in ApoE3 astrocytes (Figure 1A,B). In agreement with our data, significantly reduced ApoE levels were also observed in hiPSC-derived ApoE4 astrocytes [15,16,24], as well as in brain homogenates from mouse models expressing the human ApoE4 isoform [43,44]. It has been hypothesized that ApoE4 levels may be lower due to rapid degradation [45], but the exact mechanisms of this degradation are not yet clear. However, it appears that ApoE can be degraded by autophagy and by an LC3-associated endocytosis-like process, and that ApoE4 is sequestered in enlarged endosomes [46]. On the other hand, although previous studies reported for reduced ApoE secretion by hiPSC-derived ApoE4 astrocytes [15,16,24], in our experimental model we observed a significantly increased ApoE secretion in the ACM of ApoE4 astrocytes (Figure 1B). The evident increase in secreted ApoE could be due to a higher number of ApoE molecules associated with each ApoE-containing lipoprotein in ApoE4 ACM, as observed by Gong and colleagues [47]; however, it is still not clear the exact stoichiometry of ApoE per ApoE-lipid particle, or if it is affected by the ApoE genotype [29]. The observed increase in secreted ApoE may be also due to a reduced ApoE re-uptake through LDLR, LRP1, and ApoER2 [48] caused by the low levels of these receptors found in ApoE4 astrocytes (Figure 5); this is suggested by the increase in ApoE cerebrospinal fluid (CSF) levels observed in LDLR^-/-^ mice [49], and by the increase in ApoE released in ACM by LDLR^-/-^ primary astrocytes [50].

The maintenance of cholesterol homeostasis in the brain is essential for neuronal functioning and the abnormal brain cholesterol metabolism is associated with neurodegenerative diseases, such as AD [25]. In order to have a broader view of the lipid-related gene expression in ApoE3 and ApoE4 cell lines, the expression levels of more than forty genes were analysed and lots of them resulted up- or down-regulated in ApoE4 astrocytes. In particular, among the identified differentially expressed genes, there are genes involved in the regulation of cholesterol synthesis (e.g., SREBF1, INSIG1, HMGCS1), oxidation (e.g., CYP27A1), and efflux (e.g., ABCA1). Interestingly, LXRα and PPARγ, key proteins involved in the regulation of lipid homeostasis [51,52], have been shown to be two of the most down-regulated genes (Figure 2). These results, together with those obtained by transcriptomic and proteomic analyses of hiPSC-derived astrocytes [15,16], further confirm that lipid metabolism is one of the main altered processes in ApoE4 astrocytes.

Brain cholesterol homeostasis is highly regulated to ensure steady levels of the sterol, and it is almost entirely independent from that of the periphery because of the limited exchange between the central nervous system (CNS) and peripheral circulation due to the presence of the blood–brain barrier (BBB). Thus, cholesterol in the brain derives almost exclusively (>95%) from local de novo synthesis by astrocytes from Acetyl-CoA through reactions catalyzed by over 20 enzymes. As shown in Figure 3A, in an early stage of cholesterol biosynthesis Acetyl-CoA is converted into the first sterol, lanosterol. The first rate-limiting enzyme involved in this stage is HMGCR, which catalyzes the reduction in HMG-CoA to mevalonate giving rise to a series of reactions that lead to the formation of lanosterol. Then, in the so-called “post-lanosterol stage”, sterol intermediates proceed through either the Bloch or the Kandutsch–Russell pathway, which both ultimately lead to cholesterol synthesis. The enzyme DHCR24 may act on any intermediate in the Bloch pathway and deflect it in the Kandutsch–Russell one [53]. Concerning astrocytes, they have been shown to mainly contain precursors of the Bloch pathway [54]. In order to maintain brain cholesterol homeostasis, excess cholesterol is stored in the endochylema after esterification, or it is oxidized and converted into oxysterols (Figure 3A). The main oxysterol involved in this process is 24-OHC, also called cerebrosterol, which derives from cholesterol oxidation by CYP46A1, a cytochrome P-450 enzyme mainly expressed by neurons but also by astrocytes [55]. The 24-OHC diffuses across the BBB into the systemic circulation (∼99%) driven by the concentration gradient, and it is finally metabolized in the liver [56,57]; less than 1% of 24-OHC flows into the CSF [58]. Moreover, 24-OHC, being an LXR ligand, regulates the expression and synthesis of the proteins involved in cholesterol synthesis and efflux, including ApoE and ABCA1/ABCG1 [59]. To a lesser extent, cholesterol is oxidized into 27-OHC by CYP27A1, although most of it comes from the systemic circulation [11,14]. In addition to these two oxysterols, others are produced via enzymatic reactions, such as 25-OHC and 7α-OHC deriving from cholesterol oxidation by CH25H and CYP7A1, respectively; however, these oxysterols may be also generated by cholesterol non-enzymatic oxidation mediated by reactive oxygen species (ROS). In fact, cholesterol autooxidation can also occur, induced by different compounds such as lipid peroxides, free radical species, and metal cations, giving rise to the formation of other oxysterols, such as 7-KC and 7β-OHC [60].

In the adult brain, mature neurons mostly depend on the rate of cholesterol supplied by astrocytes. Once synthesized by astrocytes, cholesterol is loaded into lipoproteins similar to high-density lipoproteins (HDL) containing ApoE, and then it is secreted into the extracellular matrix via ABCA1 and ABCG1; then, lipoproteins are transported to neurons where they are internalized through the LDLR and LRP1 placed on the nerve cell membrane. Following receptor-mediated endocytosis ApoE is recycled, and cholesterol is used for cell membrane turnover and repair, dendritic growth, myelin formation, synaptogenesis, and neurotransmitter release [61,62]. However, LDLR, LRP1 and ApoER2 receptors are also present in astrocytes [15,24,63] and have been also shown to be involved in the Aβ clearance mediated by astrocytes [50,64].

It has been shown that metabolite levels and gene expression associated with cholesterol de novo biosynthesis are reduced in AD brain regions vulnerable to AD pathology, probably due to impaired enzymatic cholesterol catabolism and efflux [25]. Recently, it has been shown that cholesterol homeostasis is also impaired in ApoE4 astrocytes, although controversial data concerning intracellular and secreted cholesterol levels are present in literature depending on the different experimental model employed [15,16,24]. Our analysis by GC-MS pointed out that total cholesterol levels are slightly but significantly reduced in ApoE4 astrocytes in comparison to ApoE3 astrocytes (Figure 3B), similarly to what was observed by de Leeuw and colleagues in hiPSC-derived ApoE4 astrocytes [24]. The modest reduction in the amount of intracellular cholesterol could be due to the reduction in the expression levels of the two key enzymes involved in cholesterol biosynthesis, i.e., HMGCR and DHCR24 (Figure 3C), also observed in AD brain samples [25]. Moreover, the analyses pointed out that the levels of the cholesterol precursors lanosterol and lathosterol are much higher in ApoE4 astrocytes (Figure 3B), indicating a possible feedback regulation to compensate for reduced cholesterol. Conversely, desmosterol levels are lower in ApoE4 compared to ApoE3 astrocytes (Figure 3B). Based on this evidence, it could be assumed that in ApoE4 astrocytes cholesterol anabolism shifts from the Bloch pathway to the Kandutsch–Russell pathway, as suggested by desmosterol decrease and lathosterol increase. Therefore, the presence of ApoE4 genotype might affect one or more enzymes involved in the post-lanosterol phases of cholesterol synthesis. As a consequence of the Bloch-pathway inhibition, lanosterol enhances; to avoid accumulation of the latter, both the switch to the Kandutsch–Russell pathway and a feed-back HMGCR downregulation could occur, resulting altogether in a moderate, although significant, cholesterol decrease. In this connection, it is recognized that HMGCR is negatively regulated by side-chain oxidized oxysterols such as 25-OHC and 27-OHC [10], which appear to augment in our cell model; this evidence points to these oxysterols as possible important co-factors in ApoE4 astrocyte cholesterol synthesis. In addition, it is worth noting that while the Bloch pathway generally appears as typical for astrocytes, microglia cells seem to preferentially resort to the Kandutsch–Russell pathway; thus, the shift to this enzymatic cascade might reflect a sort of astrocyte polarization to a cell-type more specialized in immune responses [53]. However, due to the many enzymes involved in cholesterol synthesis and to the complex regulation of this process, a more in-depth analysis is needed to understand whether and how ApoE3 and ApoE4 astrocytes synthetize cholesterol through different pathways.

Concerning cholesterol oxidation, in our experimental model ApoE4 astrocytes have been shown to produce higher levels of the oxysterols 25-OHC and 27-OHC, but lower levels of 24-OHC, 7α-OHC and 7β-OHC compared to ApoE3 astrocytes; 7-KC levels did not change significantly (Figure 4A). Interestingly, the expression trend of the enzymes involved in the oxysterol generation goes in parallel with the levels of the corresponding oxysterol. In fact, CYP46A1 and CYP7A1 reduction is followed by 24-OHC and 7α-OHC reduction, respectively; conversely, CYP27A1 and CH25H increase is followed by 27-OHC and 25-OHC increase (Figure 4B). Considerable evidence indicates that changes in oxysterol levels in the brain could be one of the factors contributing to AD progression. Indeed, it has been demonstrated that numerous oxysterols accumulate in the brain as AD progresses (e.g., 27-OHC, 25-OHC, 7α- and 7β-OHC, and 7-KC); conversely, the levels of 24-OHC drop dramatically in the late stages of the disease likely due to neuronal loss [19,65]. Oxysterols accumulating in the brain certainly play a crucial role in AD development by enhancing oxidative stress and inflammation, with consequent neurodegeneration [11]. Notably, although most oxysterols exert detrimental effects, 24-OHC has been shown to have mainly neuroprotective properties, such as preventing tau hyperphosphorylation and Aβ production [66,67]. This leads to the likely assumption that the physiological presence of this oxysterol in the brain is fundamental to guarantee brain health, suggesting the importance of preventing its loss in the brain during the course of AD [67]. Results showed that the changes in oxysterol levels in ApoE4 astrocytes somehow reflect some of those occurring in the AD brain (i.e., 24-OHC decrease, 27-OHC and 25-OHC increase) [19]; in fact, it should be considered that other ApoE4 cell types (i.e., neurons, microglia) could differently contribute to the brain oxysterol content, as well as other systemic and/or environmental factors, among which oxidative stress. Data from this study might highlight another feature of ApoE4 astrocytes that could further explain ApoE4 AD-promoting role by affecting the levels of cholesterol oxidized derivatives. In particular, we observed a reduction in both autoxidation (i.e., 7β-OHC) and enzymatic (i.e., 7α-OHC and 24-OHC) products which, at least in part, could be due to a less availability in ApoE4 astrocytes of the oxysterol precursor, namely cholesterol itself. On the other hand, the oxysterols of enzymatic origin 25-OHC and 27-OHC are strikingly enhanced; these data indicate that factors, such as gene modulation, could affect the formation of these oxysterols more than cholesterol amount, as suggested by CH25H and CYP27A1 significant up-regulation. In this connection, it is interesting to note that while 7α-OHC, 7β-OHC, and 24-OHC are primarily involved in cholesterol homeostasis regulation, 25-OHC and 27-OHC are signalling molecules also involved in anti-viral, immunomodulant and inflammatory responses [20], further suggesting an ApoE4 astrocyte differentiation into a microglia-like phenotype, more involved in immune response than in cholesterol supply for neuronal function and development.

One of the main proteins responsible for ApoE lipidation in the CNS is the transporter ABCA1. The reduced ABCA1 protein levels observed in immortalized ApoE4 astrocytes (Figure 5) further confirm results obtained in mouse primary and hiPSC-derived ApoE4 astrocytes [68]. This marked reduction may be due to the down-regulation of the expression levels of the nuclear receptor LXRα (Figure 2), a key regulator of ABCA1 expression [51]. It has also been suggested that ApoE4 is able to alter ABCA1 protein levels by trapping it in the late-endosomes, thus reducing its recycling to the astrocytic membrane [68]. Furthermore, ABCA1 reduction is considered one of the reasons why ApoE4-containing lipoproteins are poorly lipidated [69,70]; indeed, the treatment of different mouse models expressing ApoE4 with the ABCA1 agonist CS-6253 or the LXR agonist T0901317 have been shown to increase ApoE4 lipidation, as well as to reduce ApoE4-driven Aβ accumulation and cognitive deficits [71,72].

ApoE is able to bind various receptors such as LDLR, LRP1, and ApoER2, through which ApoE-containing lipoproteins are taken up by cells [9]. Data showed that ApoE4 astrocytes contain significantly lower levels of all three receptors (Figure 5), although conflicting data regarding some of them have been obtained in primary and hiPSC-derived ApoE4 astrocytes [15,24,73]. As previously discussed, the pronounced reduction in these receptors in ApoE4 astrocytes could be one of the reasons for the increased ApoE levels detected in ACM (Figure 1), as suggested by other studies [49,50]. Concerning the impact of the ApoE genotype on the three receptors, several studies showed that ApoE4 is able to reduce their recycling to the cell membrane, likely because of the aberrant endosomal acidification that traps receptors within the intracellular compartments and leads to their degradation [73,74,75]. Moreover, astrocytic LDLR and LRP1 have been shown to be involved in Aβ uptake and clearance, as confirmed by the significant change in Aβ accumulation observed in experimental models characterized by the knockdown or the overexpression of one of these two receptors [50,64,76]; thus, studies suggest that lower levels of ApoE receptors in the astrocytic membrane may lead to the reduced ability of ApoE4 astrocytes to clear Aβ [16,73].

Another key protein that we observed to be reduced in ApoE4 astrocytes is PPARγ (Figure 2 and Figure 5). PPARγ is a ligand-modulated transcription factor which, through the formation of heterodimers with the retinoid X receptor (RXR), regulates the expression of genes involved in lipid and glucose metabolism [52]; in addition, it is known to exert a neuroprotective and anti-inflammatory role in the CNS [77,78]. Interestingly, reduced PPARγ expression and protein levels have been observed to characterize the brain of ApoE4-TR mice [79]. Barrera and colleagues showed that PPARγ regulates the transcription of several genes involved in late-onset AD, including ApoE [80]. For instance, PPARγ has been shown to regulate LRP1 protein levels in adipocytes and hepatocytes [81,82], suggesting that the decrease in LRP1 observed in ApoE4 astrocytes may be also due to the dramatic decline in PPARγ levels that characterizes this cell line (Figure 5). In addition to LRP1, other genes are regulated by PPARγ such as ABCA1, LXRα, LPL, and GYK [52,83,84], and all were found to be down-regulated in ApoE4 astrocytes (Figure 2 and Figure 5). Thiazolidinediones (TZDs) are PPARγ synthetic ligands with insulin-sensitizing properties, used for the treatment of type 2 diabetes. Since AD is also characterized by alterations of brain insulin signaling, studies on AD mouse models and AD patients have been carried out to investigate the potential of these drugs in AD treatment; however, contrasting results have been obtained so far [85,86]. In particular, a lower effectiveness of PPARγ-targeting therapies has been observed in ApoE4 carriers and may be due to the impairment of PPARγ signaling mediated by ApoE4 through still unknown mechanisms [87]. Overall, these data suggest that PPARγ down-regulation could be one of the leading causes of the alterations in cholesterol metabolism that we observed in ApoE4 astrocytes. However, PPARγ response networks are very complex and differently regulated, depending on the specific cell type [52] and its role in the brain need to be further deepened.

## 5. Conclusions

Although we are aware of the limitations of the employed experimental model, this study highlighted new ApoE4-induced alterations that concern astrocytic cholesterol metabolism, providing support for further investigations on the specific molecular mechanisms involved and the potential consequences on AD pathology. In particular, we observed that ApoE4 astrocytes are characterized by altered expression of several genes involved in cholesterol homeostasis (e.g., synthesis, oxidation, efflux, and uptake). This wide range of differentially expressed genes leads to alterations of the levels of cholesterol precursors and total cholesterol, suggesting a potential shift toward the Kandutsch–Russell pathway, as well as of the levels of several oxysterols. Moreover, ApoE intracellular and extracellular content, as well as the levels of the ABCA1 transporter and the receptors LDLR, LRP1, and ApoER2 resulted to be impaired in ApoE4 astrocytes. One of the reasons for these alterations may be the significant down-regulation of LXRα and PPARγ, both key regulators of lipid homeostasis.

Based on the obtained data, and given the many roles played by astrocytes in normal neuronal functioning and the considerable impact of astrocytic alterations in AD pathology, drug development aimed at restoring cholesterol homeostasis could be effective to counteract not only AD but also other types of dementia.

## Data Availability

The data presented in this study are available in article.
